# Functional Motions of *Candida antarctica* Lipase B: A Survey through Open-Close Conformations

**DOI:** 10.1371/journal.pone.0040327

**Published:** 2012-07-10

**Authors:** Mohamad Reza Ganjalikhany, Bijan Ranjbar, Amir Hossein Taghavi, Tahereh Tohidi Moghadam

**Affiliations:** 1 Department of Biophysics, Faculty of Biological Sciences, Tarbiat Modares University, Tehran, Iran; 2 Department of Nanobiotechnology, Faculty of Biological Sciences, Tarbiat Modares University, Tehran, Iran; King's College, London, United Kingdom

## Abstract

*Candida antarctica* lipase B (CALB) belongs to psychrophilic lipases which hydrolyze carboxyl ester bonds at low temperatures. There have been some features reported about cold-activity of the enzyme through experimental methods, whereas there is no detailed information on its mechanism of action at molecular level. Herein, a comparative molecular dynamics simulation and essential dynamics analysis have been carried out at three temperatures (5, 35 and 50°C) to trace the dominant factors in the psychrophilic properties of CALB under cold condition. The results clearly describe the effect of temperature on CALB with meaningful differences in the flexibility of the lid region (α5 helix), covering residues 141–147. Open- closed conformations have been obtained from different sets of long-term simulations (60 ns) at 5°C gave two reproducible distinct forms of CALB. The starting open conformation became closed immediately at 35 and 50°C during 60 ns of simulation, while a sequential open-closed form was observed at 5°C. These structural alterations were resulted from α5 helical movements, where the closed conformation of active site cleft was formed by displacement of both helix and its side chains. Analysis of normal mode showed concerted motions that are involved in the movement of both α5 and α10 helices. It is suggested that the functional motions needed for lypolytic activity of CALB is constructed from short-range movement of α5, accompanied by long-range movement of the domains connected to the lid region.

## Introduction

Lipases (EC 3.1.1.3) are hydrolyzing enzymes which act on the ester bonds of carboxyl esters. They hydrolyze triacylglycerol to fatty acid and glycerol. In addition to its classic function, they also catalyze the esterification, interesterification, transesterification, acidolysis, alcohololysis and aminolysis [1]–[6].

Psychrophilic lipases catalyze the lypolytic activity at low temperatures and show fascinating features in the structure-function relationship, that are potentially important in understanding cold-adapted lypolytic mechanisms in biotechnological applications. Cold-active lipases have attracted great attention due to having variety of industrial applications, i.e. synthesis of medical, pharmaceutical and fine chemicals as well as food productions and detergents. These enzymes also offer a number of promising environmental applications in waste treatment and bioremediation of oil contaminated soil and water in the cold conditions [1], [2], [7], [8].

Several features of cold-adapted enzymes, including lipases have been investigated so far to elucidate important factors which make the structure active at low temperatures. Such factors are mainly related to variations in the structure and sequence of psychrophilic proteins, compared to their mesophilic or thermophilic counterparts [9]–[12]. Significant decrease of the arginine residue portion as compared to lysine, low proline content and increased number of glycine clustering have been observed in psychrophilic proteins through sequence comparisons. Structural investigations have revealed that the fraction of non-bonded interactions is distinctly different in psychrophilic proteins as compared to mesophilic or thermophilic counterparts. A small number of electrostatic and aromatic-aromatic interactions, small hydrophobic core and non-polar exposed surfaces accompanied with decreased number of cation-π interactions are some examples of such differences [1], [9], [11], [13]–[15].

Several reports have indicated the effect of increased global flexibility on the psychrophilic enzymes, which leads to the so called plasticity effect, facilitating the substrate’s accommodation in the active site. Although there are numerous number of reports regarding the global flexibility of cold-active enzymes [1], [14], [16]–[18], there is not enough experimental information about the local flexibility, as a more important issue. Limitations of experimental techniques are the main cause of inadequate information about the local flexibility [14], [16], [19].

*Candida antarctica* lipase B (CALB) is the most widely studied psychrophilic lipase with a great number of registered patents and various applications, which encourage utilization of the enzyme as an appropriate candidate in pharmaceutical, chemical and food industries [1].

Uppenberg et al. have solved the crystal structure of CALB at 1.55 Å resolution [20], [21]. The enzyme consists of 317 amino acid residues with a classic α/β hydrolase folding structure, three disulfide bonds and classic triad active site (Ser 105, Asp 187 and His 224) under a potential lid forming helix (α5).

Till now the existing experimental reports have addressed the structural plasticity, stability and active site properties of CALB [17], [22]–[25]. Further, theoretical and molecular dynamics simulation studies have provided some information about substrate selectivity and enantioselectivity, together with flexibility of CALB in both aqueous and organic solvents [19], [26], [27]. Skjot et al. and Ferrario et al. have addressed minor conformational changes and also the flexibility of α5 lid by molecular dynamics simulation [22], [28]. All data support the fact that CALB has a flexible short lid which is responsible for its open-closed conformations. While the enzyme has no significant interfacial activation, it has a semi-covered active site consisting of α5 (as the lid) [20], [21] and α10 (as the activation element) [22]. The interfacial activation can be explained by the opening of a lid structure of the enzyme at the oil-water interface. The two forming lids, α5 (141–147: AGPLDAL) and α10 (280–288: PAAAAIVAG), construct a narrow hydrophobic active site channel with classical catalytic residues inside.

Although extensive studies have been conducted to explain psychrophily of CALB, there is no detailed and comparative study about its cold-adapted activity and structure-function relationship at high and low temperatures. There is also lack of information about CALB’s conformational changes during lypolytic activity. Therefore, necessity of utilizing molecular dynamics (MD) simulations for tracing open-closed conformations under different temperatures comes into the picture. There have been several relevant reports about other lipases so that the mechanism of structural transition between open and closed conformations has been investigated by MD simulation, such as thermoalkalophilic lipase T1 [29], *Pseudomonas aeruginosa* lipase [30], *Candida rugosa* lipase, *Bacillus subtilis* lipase [31]. *Burkholderia cepacia* lipase [32], [33], *Yarrowia lipolytica* lipase [34], *Rhizomucor miehei* lipase, and *Thermomyces lanuginose* lipase [35].

**Figure 1:**
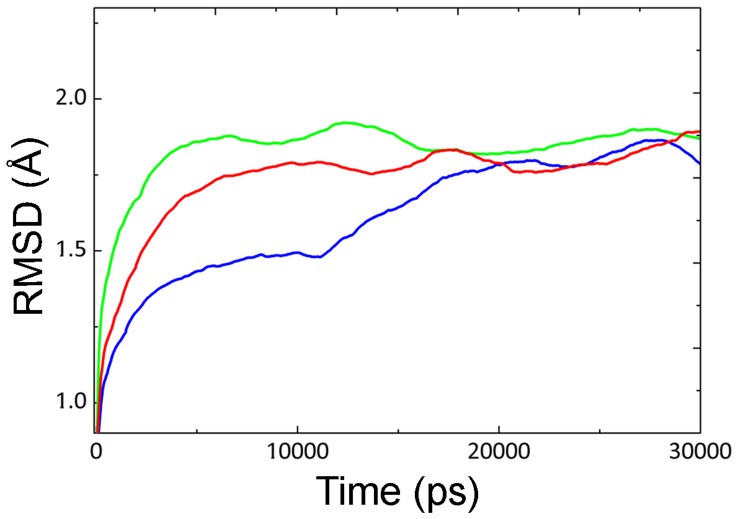
All-atom RMSD of CALB at different temperatures. All-atom RMSD of CALB at 5°C (black line), 35°C (red line) and 50°C (green line).

**Figure 2:**
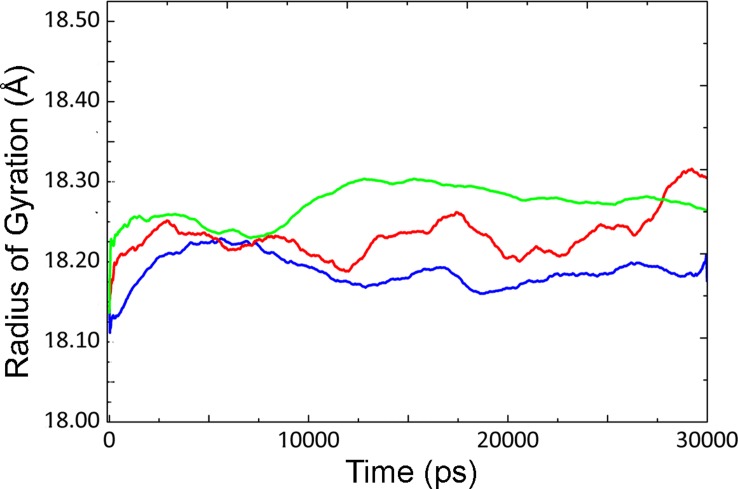
Radius of gyration of CALB at different temperatures. R_gyr_ of CALB at 5°C (blue line), 35°C (red line) and 50°C (green line) with average values of 18.188, 18.22 and 18.251 respectively.

Normal mode analysis (NMA) is a powerful method used to investigate collective motions and large-scale conformational transitions in proteins. Apart from localized motions (detected by MD simulation), large-scale motions (detected by NMA) also play important role in the functional motions of an enzyme. There have been several reports for solving complex conformational transitions. In this regard, symmetric breathing mode in the tE2 component of pyruvate dehydrogenase complex [36], ATP synthase F1-ATPase [37], ribosome movement [38], conformal changes in GroEL [39], opening of membrane channel [40] are a number of examples for NMA application.

Herein, MD simulation and NMA have been undertaken as powerful techniques to probe the local flexibility and large-scale motions of CALB. Results of the study clearly revealed that temperature does affect the enzyme’s flexibility with meaningful differences in the lid region. To achieve open-closed conformations, different sets of long-term simulations (60 ns) were performed at low, medium and high temperatures (i.e. 5°C, 35°C and 50°C). The effect of temperature was also investigated using radial distribution function of water molecules in the active site. Normal mode analysis also verified the flexibility of the lid region and revealed clear movement of domains forming the active site cleft along the low-frequency modes.

**Figure 3:**
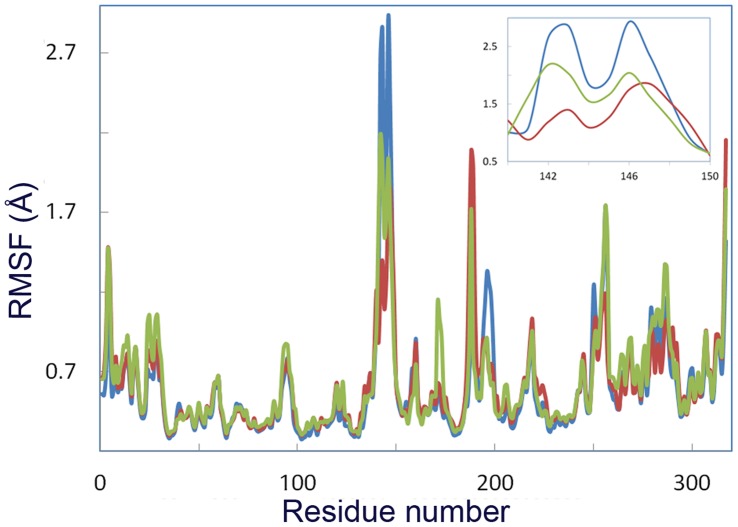
RMSF of C_α_ atom of CALB at different temperatures. RMSF of C_α_ atom of CALB at 5°C (blue line), 35°C (red line) and 50°C (green line).

**Figure 4:**
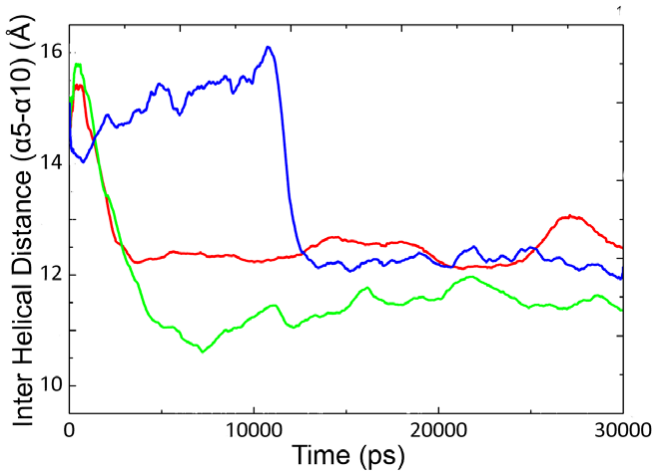
Distances between lid α5 and α10 during 30 ns. Inter helical distance between C_α_ atoms of α5 and α10 in CALB at 5°C (blue line), 35°C (red line) and 50°C (green line) as a function of time (ps).

## Results

### Structural Investigation

Figures 1 and 2 depict root mean square deviation (RMSD) and radius of gyration (R_gyr_) of CALB at different temperatures (during 30 ns), as a measure of global stability. The RMSD value of CALB increased with the concomitant rise of temperature from 5°C to 50°C. Increase in the RMSD value at 5°C is clearly visible at 12 ns, which could be due to conformational changes of the structure. Slight increase was observed for R_gyr_ as temperature raised from 5°C to 50°C. Similar trend is visible for the general behavior of RMSD and R_gyr_ as the temperature rises from 5°C to 35°C and then to 50°C (see also Figures S1 and S2).

**Figure 5:**
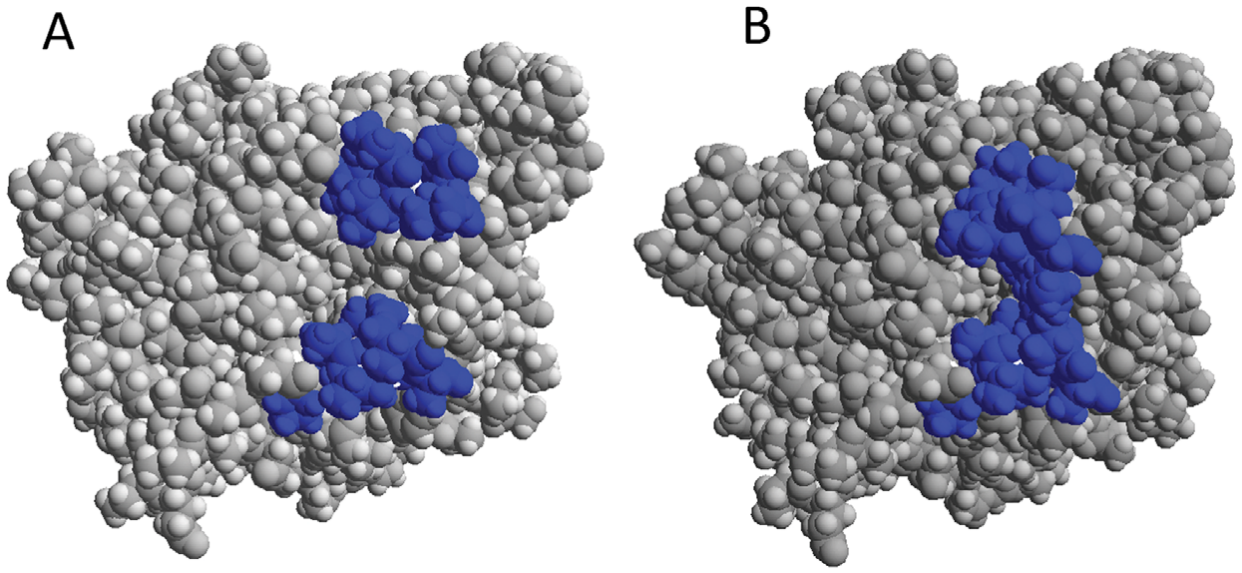
Space filling representation of CALB at open and closed conformations. Space filling representation of CALB. Average structures during 7–9 ns (left) and 12–14 ns (right) of simulation with the two highlighted helices where upper helix is shown as α5 and lower helix as α10.

**Table 1:** Cα displacement in open/closed conformations.

Residue Number	Displacement (Å)
Pro 143	7.55
Leu 144	4.38
Asp 145	5.05
Ala 146	8.19
Leu 147	5.96

Root mean square fluctuations (RMSF) of C_α_ (Figure 3) revealed significant local flexibility with distinct differences at 5, 35 and 50°C, for the residues 142–147, which are located at the α5 helix. The extent of flexibility at α5 is much higher at 5°C rather than other temperatures. Flexibility of α10 helix is increased with a lower extent as the temperature raised. For residues 184–192, amongst which Asp 187 is located, the extent of flexibility is much higher at 35 and 50°C, compared to that of 5°C.

**Figure 6:**
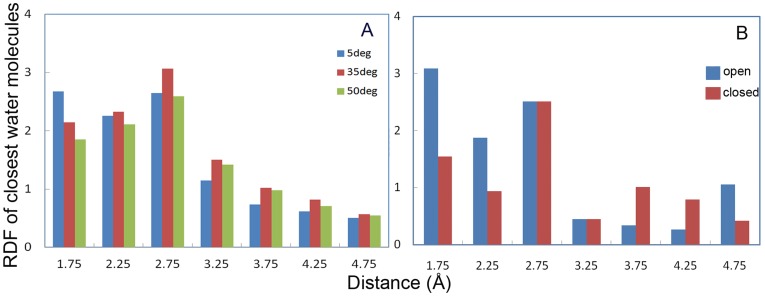
Radial distribution function of water at active site region. Average RDF of water molecules with respect to CALB active site (Ser 105) at 5°C (blue), 35°C (red) and 50°C (green) (A) . Average RDF of water molecules regarding to the CALB active site (Ser 105) on the open conformation during 7 −9 ns (blue) and closed conformation during 12–14 ns (red) (B).

**Figure 7:**
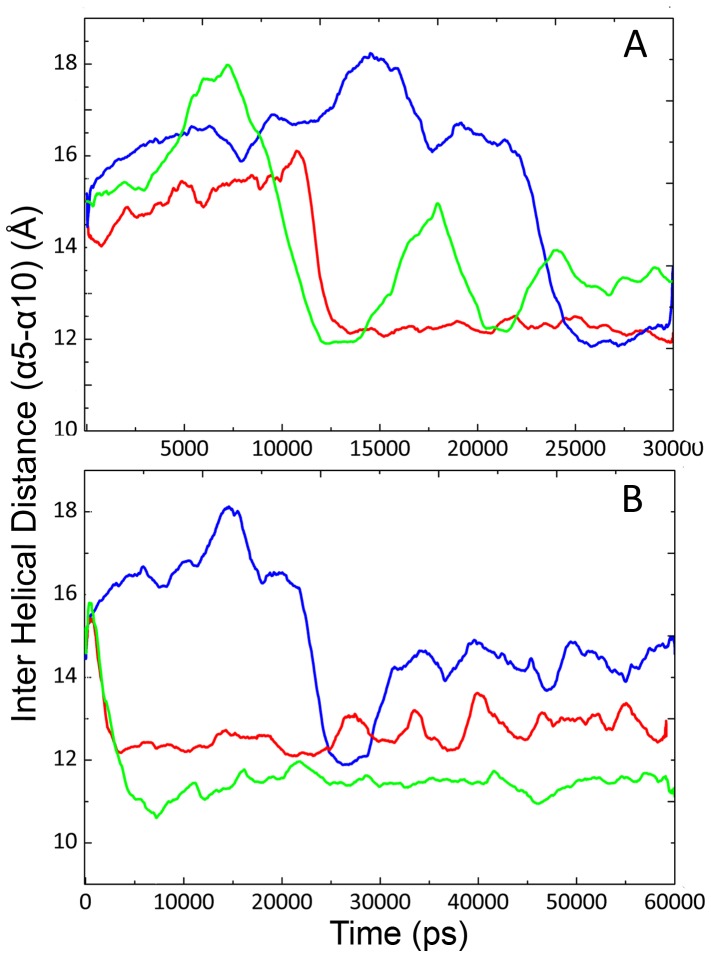
Distances between lid α5 and α10 at different sets of simulations. Inter helical distance of three different simulations of CALB at 5°C for 30 ns. First experiment (blue), second experiment (red) and third experiment (green) (A). Helical distance of long–term simulation of CALB at 5°C (blue), 35°C (red) and 50°C (green) for 60 ns. The structure closed and re-opened at 23 and 31 ns of simulation sequentially at 5°C (B).

**Figure 8:**
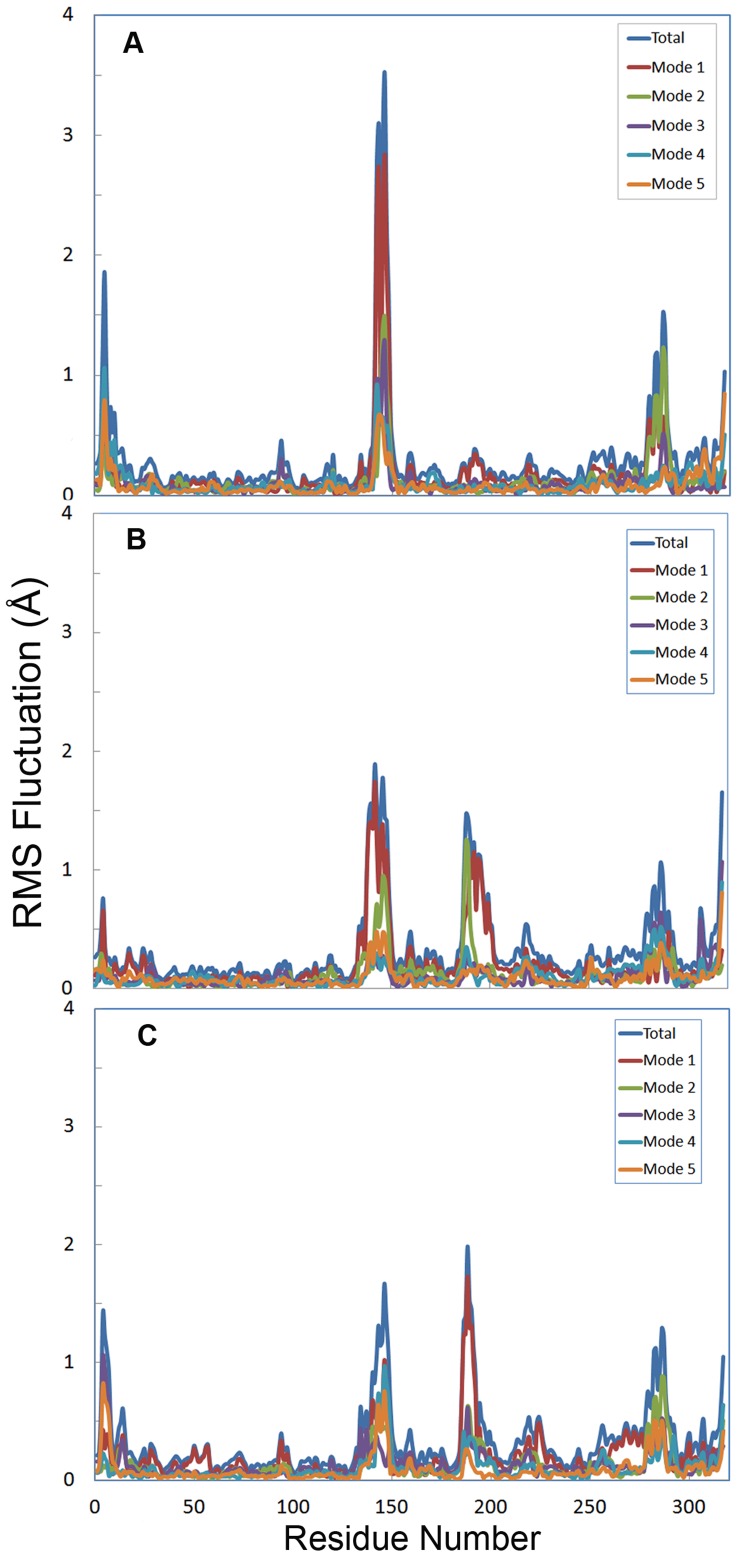
RMSF of Cα for modes 1–5 at different temperatures. RMSF of Cα for modes 1–5 at 5°C (8A), 35°C (8B) and 50°C (8C): Total RMSF (blue), mode 1 (red), mode 2 (green), mode 3 (purple), mode 4 (light blue), mode 5 (orange).

**Figure 9:**
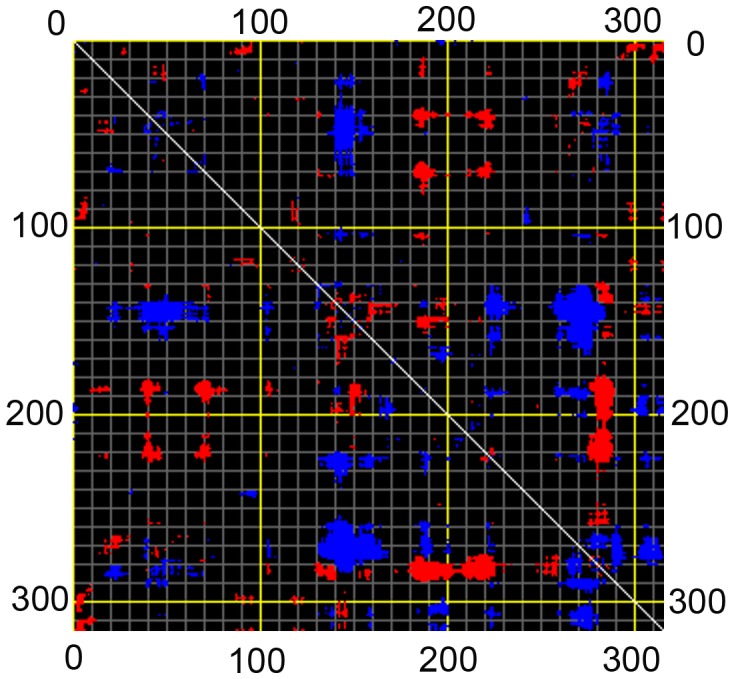
Distance fluctuation map of CALB. Distance fluctuation map of CALB for Cα at mode 1. Flexible blocks are colored for increase (blue) and decrease (red) of distance fluctuations.

**Figure 10:**
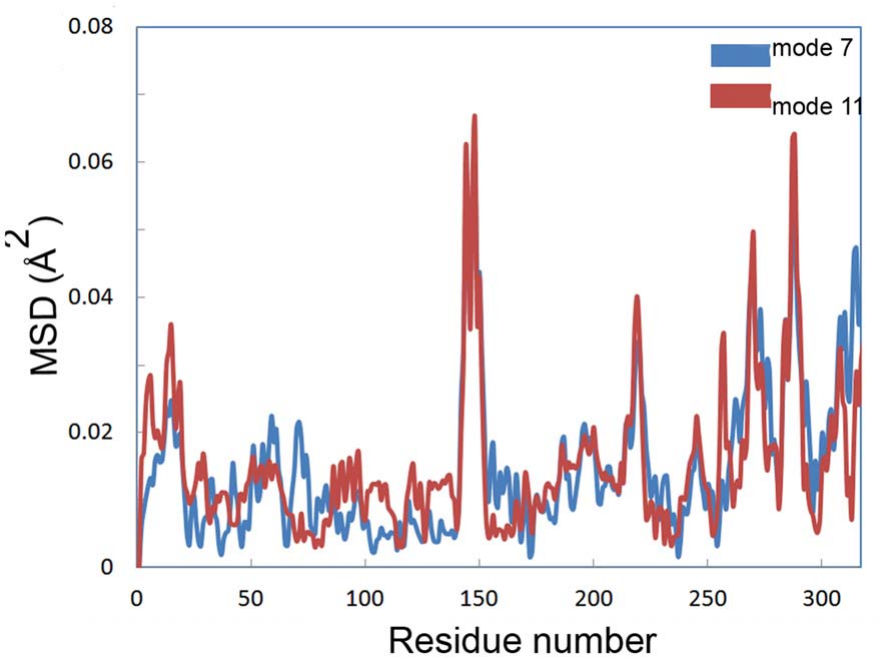
Mean square displacement of Cα. Mean square displacement of Cα calculated by normal mode analysis for mode 7 (blue) and 11 (red).

### Active Site Investigation

In order to study the effect of temperature on the active site plasticity of CALB, the distance between two lid forming helices (α5 and α10) was measured as a function of time (Figure 4). Such information is suitable for the assessment of active site accessibility for a better understanding of open-closed structures. According to the previous reports, the crystal form of CALB was obtained at open conformation with inter helical distance (α5–α10) of ∼15 Å. In this study the distance between two helices decreased immediately at 35°C and 50°C, while it remained stable at 5°C for long simulation period (0–12 ns). It could be suggested that such distance alterations are responsible for inducing conformational changes in the lid region (see also Figure S3).

Several snapshots were extracted from trajectories at 5°C during the 30 ns simulation and the average structure of CALB from 7–9 ns and 12–14 ns were superimposed. Two distinctly different conformations were observed, suggesting open-closed conformations. Figure 5 shows the average structure of CALB during 7–9 ns and 12–14 ns of simulation representative for open and close conformation respectively.

**Figure 11:**
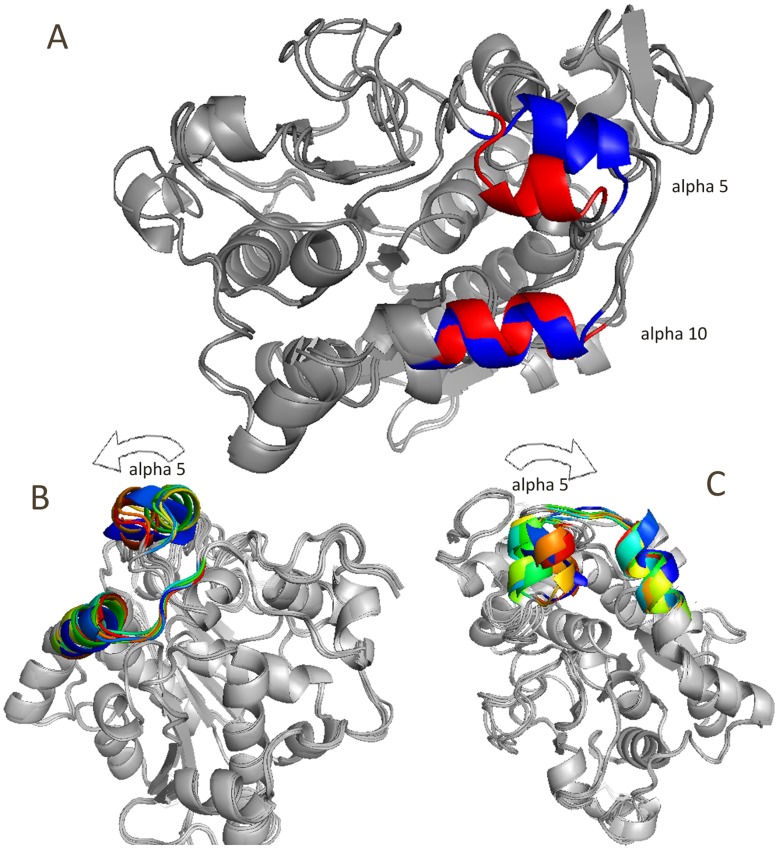
Ribbon representations of CALB. Ribbon representations of CALB with open (blue) and closed (red) conformations (A). Snapshots from different views (B and C).

**Figure 12:**
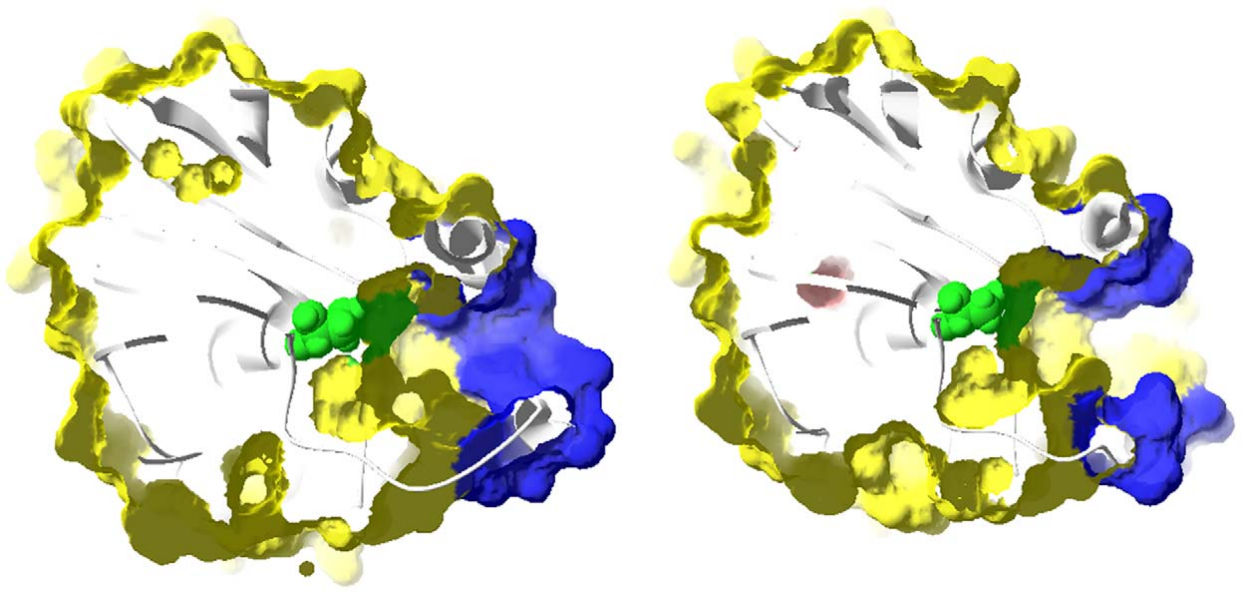
Slab view of surface representation of CALB in the active site region. Slab view of surface representation of CALB in the active site region at open (right) and closed (left) conformations. The active site (Ser 105) is shown in green.

**Figure 13:**
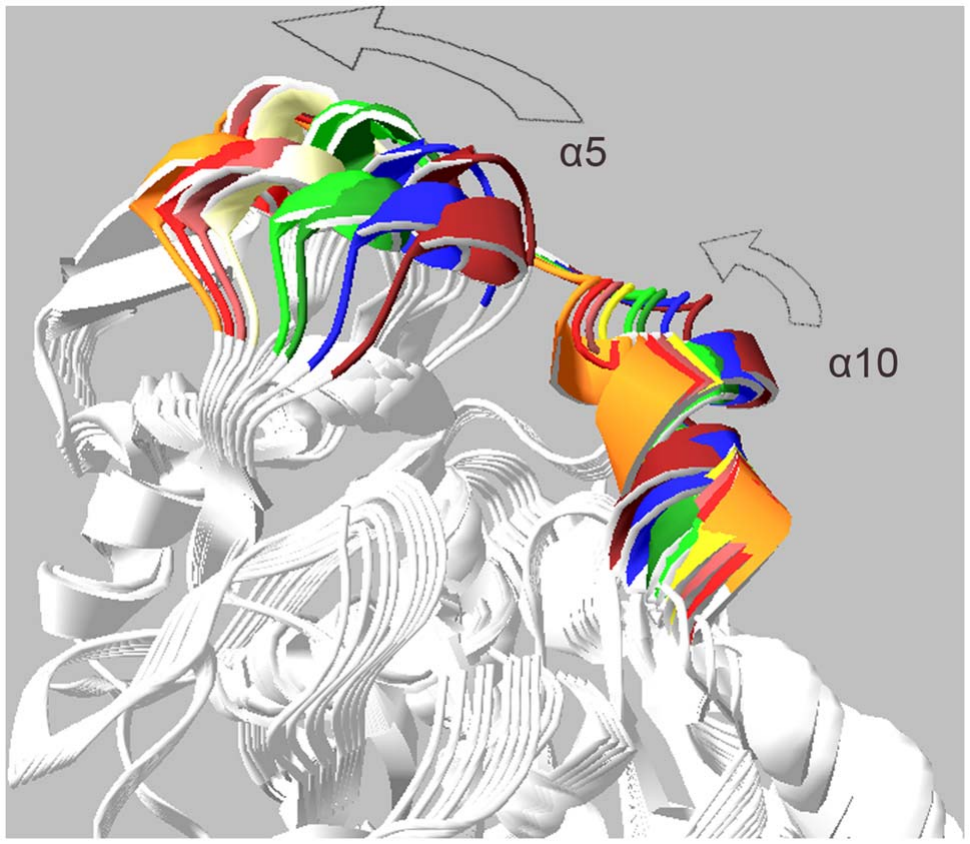
Structure snapshots of normal mode analysis. Superimposed snapshots of the structure extracted from normal mode analysis. Alpha 5 and alpha 10 helices are colored.

The displacement of C_α_ residues 143–147 were calculated for closed form, with respect to the open conformation (Table 1). It was noticed that α5 has significantly shifted toward the active site cleft, accompanied by the complete movement of its side chains into the channel, where it occupies the opening of the closed conformation. No reorientation has been observed for α10 helix except for its N-terminal side which is slightly moved toward the active site cleft.

Radial distribution functions (RDF) of the closest water molecules with respect to the active site residue (Ser 105) were calculated at different temperatures, which are interestingly in accordance with our previous results about the active site plasticity at low temperatures (see Figure 3). RDF of water molecules with respect to the active site cavity clearly showed that the probability of water molecules being close to the Ser105 is higher at 5°C rather than other temperatures (Figure 6A). RDF of water molecules was also calculated at 5°C at the suggested open and close conformations during 7–9 and 12–14 ns of simulation, respectively. As depicted by Figure 6B, the RDF value of water molecules in the open conformation is significantly higher than that of the closed one.

**Figure 14:**
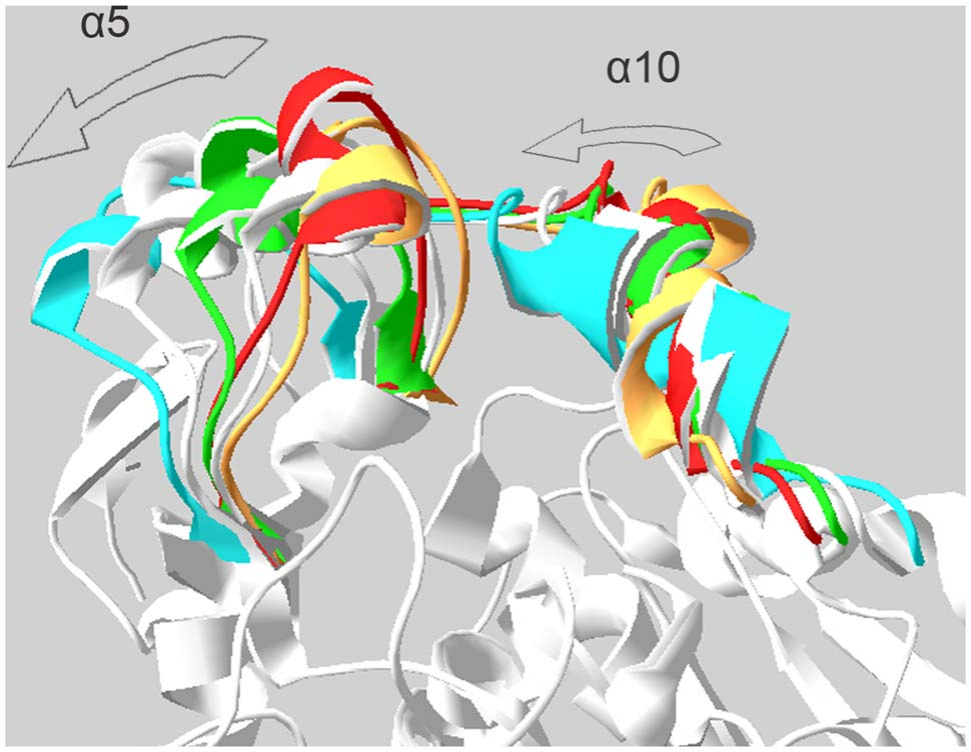
Superposition of MD and NMA results. Superposition of structures resulted from MD and NMA. Alpha 5 and 10 helices are highlighted. Closed and open conformations extracted from MD trajectories are represented by red and green.

### Extension and Reproduction of Simulations

To verify the structural alterations at 5°C, two long-term simulations with random seed number were performed again. Inter helical distances of three simulations signify the same trend, as the lid remained open for a considerable simulation time from the beginning (Figure 7A).

In order to obtain sequential conformational changes, the simulation time was extended up to 60 ns for 5, 35 and 50°C (Figure 7B). To verify the results, extension of simulations were performed for two times with different initial velocities from 30 ns up to 60 ns. The inter helical distance increased at 5°C, which signifies the formation of open structure at 31 ns. At this temperature, CALB’s structure undergoes sequential close and open conformations at 23 ns and 31 ns, respectively. Value of the helical distance remained constant at around 15 Å in the last 29 ns of simulation. In the case of 35 and 50°C, there was an abrupt decrease in the helical distance. In contrast to 5°C, the closed conformation remained intact for both 35 and 50°C during 60 ns of simulation (see also Figures S4 and S5).

### Essential Dynamics Analysis

Principal component analysis (PCA) of CALB has been performed at different temperatures. The first five collective modes of fluctuations are shown in Figure 8. RMSF for modes 1–5 at 5°C, 35°C and 50°C are depicted in Figure 8A, 8B and 8C, respectively. As clearly shown, for 5°C the extents of flexibility along five modes are remarkable in α5 and α10 helices, whereas it decreases upon increase of temperature (35 and 50°C). An increase in the flexibility of residues 183–208 (a loop connecting β6 to β7 strand) and N-terminal of the structure was observed at 35 and 50°C (see also Figures S6, S7 and S8).

### Normal Mode Analysis

Distance fluctuation maps were calculated for modes 7–12 to define the strongest variations in the Cα distance (Figures 9, S9 and S10). According to the map of mode 7, the topmost flexible elements are located in 135–155 and 280–290 regions, covering α5 and α10 helices, respectively. Mean square displacement of Cα at modes 7 and 11 showed that the extent of displacement is much considerable at mentioned elements rather than other part of the structure (Figure 10).

## Discussion

RMSD values at 5°C showed an obvious increment at 12 ns of simulation (Figure 1) with a simultaneous decrease of helical distance (Figure 4). Further investigation of the trajectories at different long-term simulations for 5°C, revealed a distinct reorientation in the lid forming helix (α5), suggesting an exchange in the open-closed conformations. Upon such structural alterations, the active state might turn into the inactive form (Figure 7).

Relevant simulation studies have shown that several lipases tend to stay in closed state in aqueous medium even though the starting structure is in an open state at 300 and 310 K [32], [33], [35]. It was also observed that such lipases remain in open state or become open from the closed state in organic solvents. This behavior was concluded as the unfavorable interaction of interior hydrophobic active site with water molecules, while it becomes more favorable in organic solvents. In other words, the energy barriers for transition between two conformations are lowered at organic medium [32].

Similar observations have been mapped in our results, as CLAB tends to be in closed state at medium and high temperatures. In contrast, CALB stays in open conformation for a long time and even comes back to open conformation after that of closed state.

According to the previous reports about cold denaturation of proteins, as temperature decreases, hydration of tightly packed hydrophobic area of protein becomes more favorable thermodynamically, making the internal non-polar groups more exposed to water [14], [41], [42]. Such phenomenon is observed at super cooled conditions where the protein becomes denatured. Cold activity mostly occurs at temperatures above 0°C for cold-adapted enzymes. The small hydrophobic core inside of such enzymes with exposed hydrophobic residues on the surface [13], [14], [43] provide an optimum dynamics and flexibility that makes them turn into cold viable structures.

In the case of CALB, there exists a very hydrophobic active site surrounded by a large hydrophobic surface around 450 Å^2^ [21]. The two forming lids, α5 and α10 consist of a delicate balance between small non-polar amino acids and proline.

As the most probable reason, it could be suggested that the architecture of the active site region (hydrophobicity and flexibility) plays important role in the cold-activity of CALB, enabling the lid to have optimal dynamics at low temperature. While such flexible parts help CALB to change its conformation from close to open easily, hydrophobicity (required for lipid-active site interactions) helps the lid to be more energetically favorable to flap at low temperature [44].

Uppenberg et al. have shown that the removal of β-octyl glucoside (as a ligand) altered α5 to a disordered structure in the open conformation in monoclinic crystal form of CALB [20]. The same observation is mapped in our study, since α5 is clearly changed to a semi-disordered structure through different sets of simulations and temperatures. Such semi-disordered structure was observed for the closed conformation with a decrease in the helical distance. Rehm et al. and Trodler et al. reported similar results in *Candida rugosa* lipase, *Rhizomucor miehei* lipase, *Burkholderia cepacia* lipase and *Thermomyces lanuginose* lipase through molecular dynamics simulation in water [33], [35].

To distinguish between an irreversible structural movement (caused by trapping the structure in a misfolded conformation) and a dynamic movement with a sequential open-closed conformational exchange, MD simulation was prolonged to 60 ns. After a decrease in the inter helical distance at 23 ns, opening of the lid was clearly observed at 31 ns of simulation, indicating that open-close reorientations can be originated from a continual structural exchange (as an enzymatic breathing) which is necessary for enzyme activity (see Video S1). Probability of the presence of water molecules in the active site cleft also confirmed such reorientations, being in accordance to the previously reported results.

Although CALB is not classified as a true lipase due to not having interfacial activation [21], still it shows structural changes during the lypolytic activity. Two models of open and closed conformations for lipase B from *Candida antarctica* have been depicted as ribbon model (Figure 11), justifying the variation between two distinct conformations (see also Figures S11 and S12).

An insight into the active site also describes a solvent accessible narrow channel with hydrophobic walls which is 10 Å × 4 Å wide and 12 Å deep, as measured from Oγ of Ser 105 [21]. Accessibility of the active site cleft shows that side chains of α5 (Leu 144 and Leu 147) occupy the entrance at the closed conformation (Figure 12). This observation is interestingly in accordance with the results reported by Uppenberg et al. (in the orthorhombic crystal form of CALB), where upon removal of the ligand, side chain of leucine points into the cleft and leads to the active site [20].

Mean square displacement graph obtained from MNA also indicated that the most moving parts of the structure are mainly located in α5 and α10 helices (Figure 10). There is an obvious conformational change related to the active site for modes 7, 9 and 11. Two moving domains were recognized which turn along two distinct axes. This leads to remarkable opening of the active site cleft. Figure 13 depicts snapshots of the structure for mode 7 (see also Video S2).

Comparison of MD and NMA results revealed that the closed state is identical for both of them. It was also observed that harmonic motion of structure results in a wider and more exposed active site (Figure 14).

To summarize, the results presented in this study suggest that psychrophily of CALB might be as the result of a delicate combination of different factors, simultaneously playing role at low temperature. Not only the sequence, but also hydrophobicity of the surface is very important, enabling the protein to have an optimized dynamics according to its functional temperature [45]. Flexibility of CALB at low temperature is localized to its active site area, while the global stability of enzyme is not affected by temperature significantly. It could be suggested that the three disulfide bonds help general stability of enzyme from being denatured at both low and high temperatures.

It can be concluded that the closed conformation is resulted by the movement of α5, accompanied by the movement of its side chains directly toward the cleft, where a part of active site is covered by structural rearrangements. According to the essential dynamics analysis for 35 and 50°C, not only the closed conformation is responsible for enzyme inactivation, but also increase of loop flexibility for residues 183–208 might justify thermo sensitivity of CALB. Considering that the loop is holding catalytic residue (Asp 187), increase in the flexibility of this region would probably disarrange the geometry of catalytic triad.

With the help of molecular dynamics simulation, as a reproducible technique, results of this investigation led to detection of CALB’s conformational changes in details, explaining the fascinating aspect of lipase activity, leaving behind the exhausting experimental procedures. Therefore, the current study revealed that opening of the lid is temperature dependent, while no external force or bonded substrate have been applied.

It is worth to mention that conclusion of this report are based upon the current observations. However, in the case of techniques availability, it would be a good idea to provide a series of experimental data to complete the last pieces of structure-function relationships of CALB puzzle. Findings of this research could be of great interest for engineering cold-adapted lipases which further encourages utilization of such enzymes with variety of industrial and medical applications.

## Materials and Methods

### Structural Preparation

Crystal structure of CALB was obtained from Protein Data Bank (1TCA) as the initial structure for MD simulations. CALB was crystallized in open conformation while no closed conformation captured in its crystal form [20], [21]. Structural investigations were performed by Swiss-PDB viewer 4.0.1 and Pymol 1.3 [46], [47]. Visual Molecular Dynamics (VMD 1.9.1) has been used to prepare animations for CALB’s structural movements [48].

### System Setup for Molecular Dynamics Simulations

Molecular dynamics simulation was performed by AMBER package (version 10) with ff99SB force field [49], [50]. The proper protonation state of active site histidine (protonated N_D_ of His224) has been adjusted and three disulfide bonds were introduced to the structure [20], [21], [26]. Structural neutralization was carried out by addition of a sodium ion to the structure. Solvation of CALB in 10 Å layer of explicit water TIP3P model were made in a truncated octahedral box, by *xLEaP* (version 1.4) and the coordination and topology files were then saved for the next steps of minimization and MD simulations.

### Energy Minimization and Molecular Dynamics Simulations

Minimization of the solvated CALB was conducted in two steps. Primarily, water and ion were minimized with 1500 steps (500 steps of steepest decent, followed by 1000 steps of conjugate gradient) then the whole system was minimized with 2500 steps (1000 steps of steepest decent followed by 1500 steps of conjugate gradient). Non-bonded interactions were calculated at cutoff distance of 10 Å by PME (Particle Mesh Ewald) method in periodic boundary conditions [51].

The system was gradually heated from 0 to 278, 308 and 333 °K for a period of 150 ps, using Langevin thermostat with collision frequency of 2 ps^−1^ [52]. Heating was done with the NVT ensemble under periodic boundary conditions. The equilibration was performed for an interval of 800 ps in the NPT ensemble, before using long-term molecular dynamics simulations. Finally, production MDs were performed for 30 ns (at 5°C, 35°C and 50°C) with the NPT ensemble. SHAKE algorithm was applied to constrain bonds involving hydrogen atoms [53]. The coordinates were then saved every 0.8 ps.

### Extension and Reproduction of Simulations

To assure the reproducibility of results, all of the simulations were repeated three times with random initial velocities. To capture and verify the sequential open-closed conformations, simulations of CALB at 5, 35 and 50°C were prolonged up to 60 ns for two times.

### Analysis of Trajectories

Trajectories were analyzed by *ptraj* (version 1.4) [54]. The root mean square deviation, fluctuation and radius of gyration were calculated according to the initial structure. In order to investigate the active site properties, the distance between secondary structural elements and RDF of closest water molecules (with respect to the active site) were calculated as two important factors. The distance between lid α5 and α10 was calculated according to backbone’s center of mass for residues 143–145 (α5 helix) with respect to residues 283–285 (α10 helix). KaleidaGraph 4.1 was used for smoothing, analysis and plotting of data obtained from RMSD, R_gyr_ and helical distances.

The essential dynamics of CALB were performed based on the diagonalization of covariance matrix, extracted from MD trajectories at different temperatures (5, 35 and 50). Overall translational and rotational motions were filtered by fitting into the reference structure. Principal component analysis was conducted for highest collective fluctuation modes (1–5). RMS fluctuations of Cα were calculated for five modes at these temperatures.

### Normal Mode Analysis

Normal mode analysis of CALB was carried out by Elastic Network Model (*ElNemo*) server [55]. Five lowest-frequency modes with a high degree of collectivity (modes 7–12) were calculated with server’s default values. Results of NMA were reported as Cα distance fluctuation maps and mean square displacement. Ten snapshots were extracted from each mode for further structural analysis.

## Supporting Information

Figure S1 **All-atom RMSD of CALB at different temperatures.** RMSD of all-atom of CALB at 5°C (blue line), 35°C (red line) and 50°C (green line). (Non-smoothed version of Figure 1)
(TIF)

Figure S2 **Radius of gyration of CALB at different temperatures.** R_gyr_ of CALB at 5°C (blue line), 35°C (red line) and 50°C (green line). (Non-smoothed version of Figure 2)
(TIF)

Figure S3 **Distances between lid α5 and α10 during 30 ns.** Inter Helical distance between C_α_ atoms of α5 and α10 in CALB at 5°C (blue line), 35°C (red line) and 50°C (green line) as a function of time (ps). (Non-smoothed version of Figure 4)
(TIF)

Figure S4 **Distances between lid α5 and α10 at different sets of simulations.** Inter helical distance (α5–α10) of three different simulations of CALB at 5°C for 30 ns. First experiment (blue), second experiment (red) and third experiment (green) (A). Inter helical distance of long–term simulation of CALB at 5°C (blue), 35°C (red) and 50°C (green) for 60 ns. Active site conformation closed and re-opened at 23 and 31 ns of simulation sequentially at 5°C (B). (Non-smoothed version of Figure 7)
(TIF)

Figure S5 **Distances between lid α5 and α10 at different sets of reproduced simulations.** Inter helical distance (α5–α10) of reproduced long–term simulations of CALB at 5°C (blue), 35°C (red) and 50°C (green) for 60 ns. Simulations have been reproduces from 30 ns up to 60 ns. Active site conformation re-opened and closed at 32 ns and 47 ns of simulation at 5°C.
(TIF)

Figure S6 **RMSF of CALB calculated from PCA at 5°C.** RMS Fluctuations of CALB calculated from PCA for modes 1–5 at 5°C. Total RMSF (1), mode 1 (2), mode 2 (3), mode 3 (4), mode 4 (5), mode 5 (6).
(TIF)

Figure S7 **RMSF of CALB calculated from PCA at 35°C.** RMS Fluctuations of CALB calculated from PCA for modes 1–5 at 35°C. Total RMSF (1), mode 1 (2), mode 2 (3), mode 3 (4), mode 4 (5), mode 5 (6).
(TIF)

Figure S8 **RMSF of CALB calculated from PCA at 50°C.** RMS Fluctuations of CALB calculated from PCA for modes 1–5 at 50°C. Total RMSF (1), mode 1 (2), mode 2 (3), mode 3 (4), mode 4 (5), mode 5 (6).
(TIF)

Figure S9 **Distance Fluctuation map calculated from NMA.** Distance Fluctuation map of mode 9 calculated from NMA.
(TIF)

Figure S10 **Distance Fluctuation map calculated from NMA.** Distance Fluctuation map of mode 11 calculated from NMA.
(TIF)

Figure S11 **Ribbon representations of CALB.** Ribbon representations of CALB at open (left) and closed (right) conformation. α5 (up) and α10 (down) are shown in blue. Semi-disordered α5 is clearly depicted in the closed conformation.
(TIF)

Figure S12 **Surface and excluded ribbon representations of CALB at open and closed conformations.** Surface representations of CALB at open and closed conformations (A and B). Excluded ribbon represents α5 and α10 in open and closed conformations (C and D).
(TIF)

Video S1 **CALB conformational changes captured by MD simulation.** Open-closed conformational changes of CALB captured by MD simulation. The looped animation depicts movement of α5 toward α10, giving the closed conformation.
(MPG)

Video S2 **CALB harmonic structural motions captured by NMA.** Harmonic structural motions of CALB captured by normal mode analysis. The looped animation depicts movements of both α5 and α10, giving the open-closed conformations.
(MPG)
