# Supplementary figures and images for: Functional Motions of Candida antarctica Lipase B: A Survey through Open-Close Conformations
Source: PLoS One. 2012 Jul 10;7(7):e40327. doi: 10.1371/journal.pone.0040327 (PMC3393743; doi:10.1371/journal.pone.0040327)

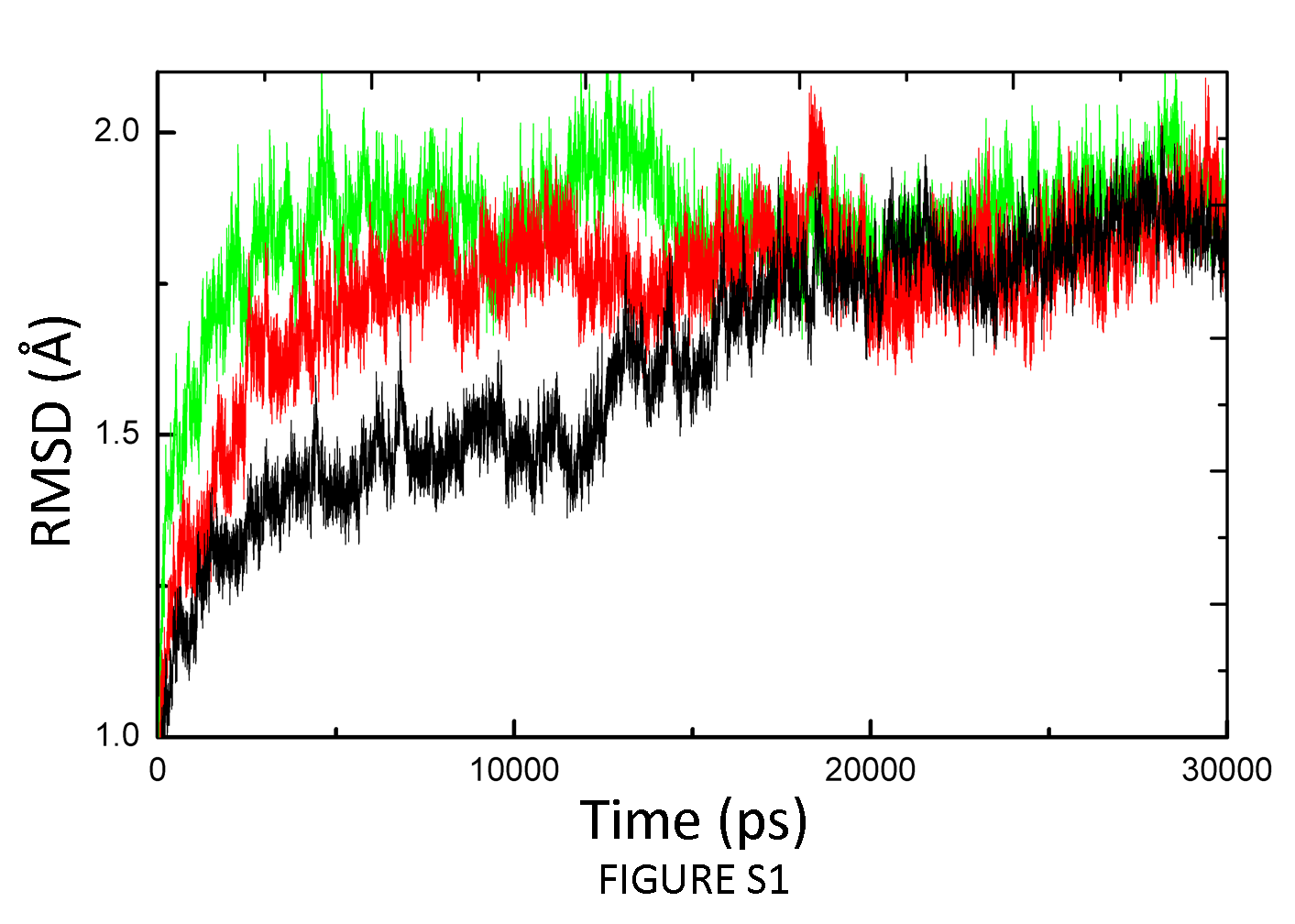

Supplement: Figure S1 — All-atom RMSD of CALB at different temperatures. RMSD of all-atom of CALB at 5°C (blue line), 35°C (red line) and 50°C (green line). (Non-smoothed version of Figure 1) (TIF) [file pone.0040327.s001.tif]

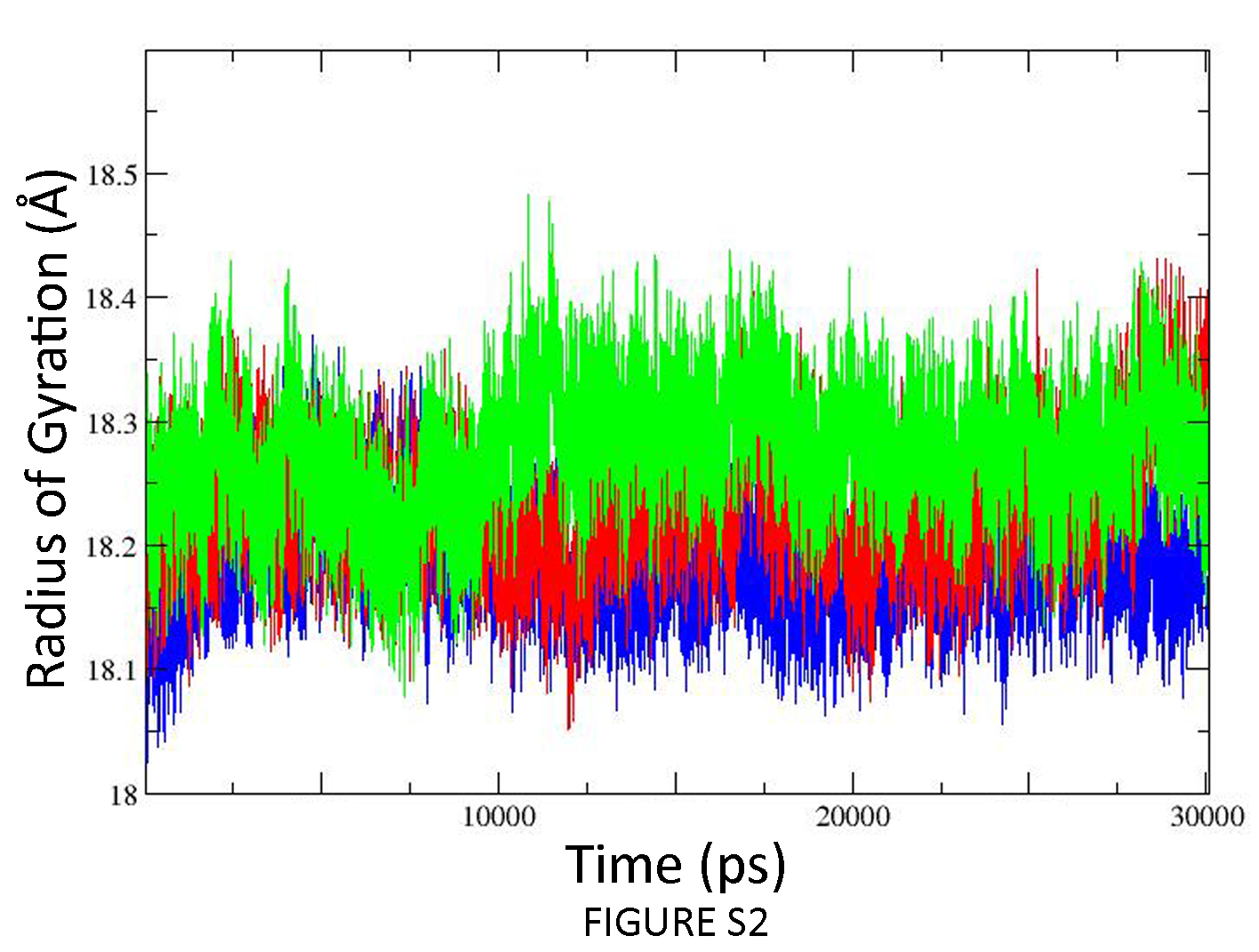

Supplement: Figure S2 — Radius of gyration of CALB at different temperatures. Rgyr of CALB at 5°C (blue line), 35°C (red line) and 50°C (green line). (Non-smoothed version of Figure 2) (TIF) [file pone.0040327.s002.tif]

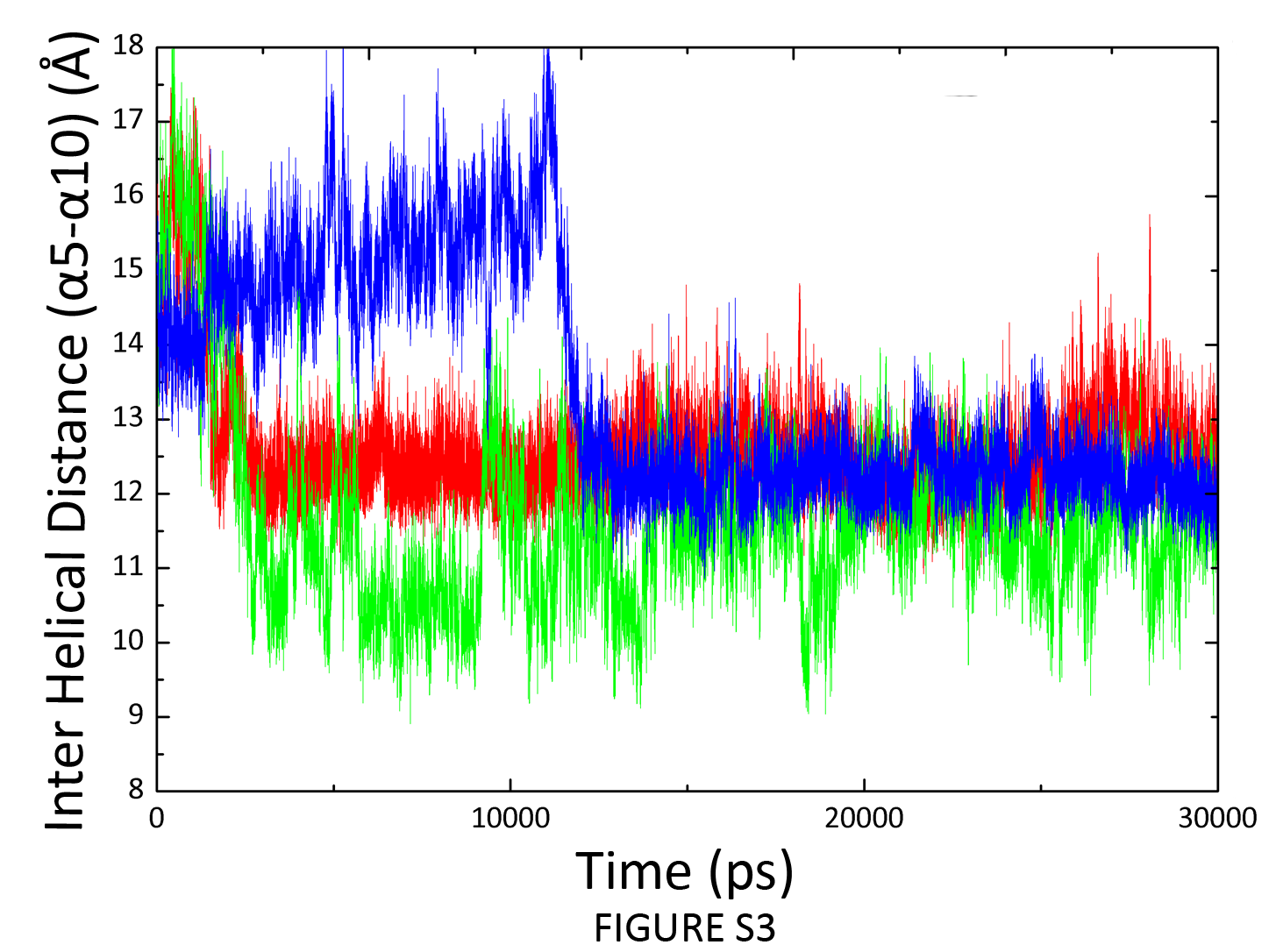

Supplement: Figure S3 — Distances between lid α5 and α10 during 30 ns. Inter Helical distance between Cα atoms of α5 and α10 in CALB at 5°C (blue line), 35°C (red line) and 50°C (green line) as a function of time (ps). (Non-smoothed version of Figure 4) (TIF) [file pone.0040327.s003.tif]

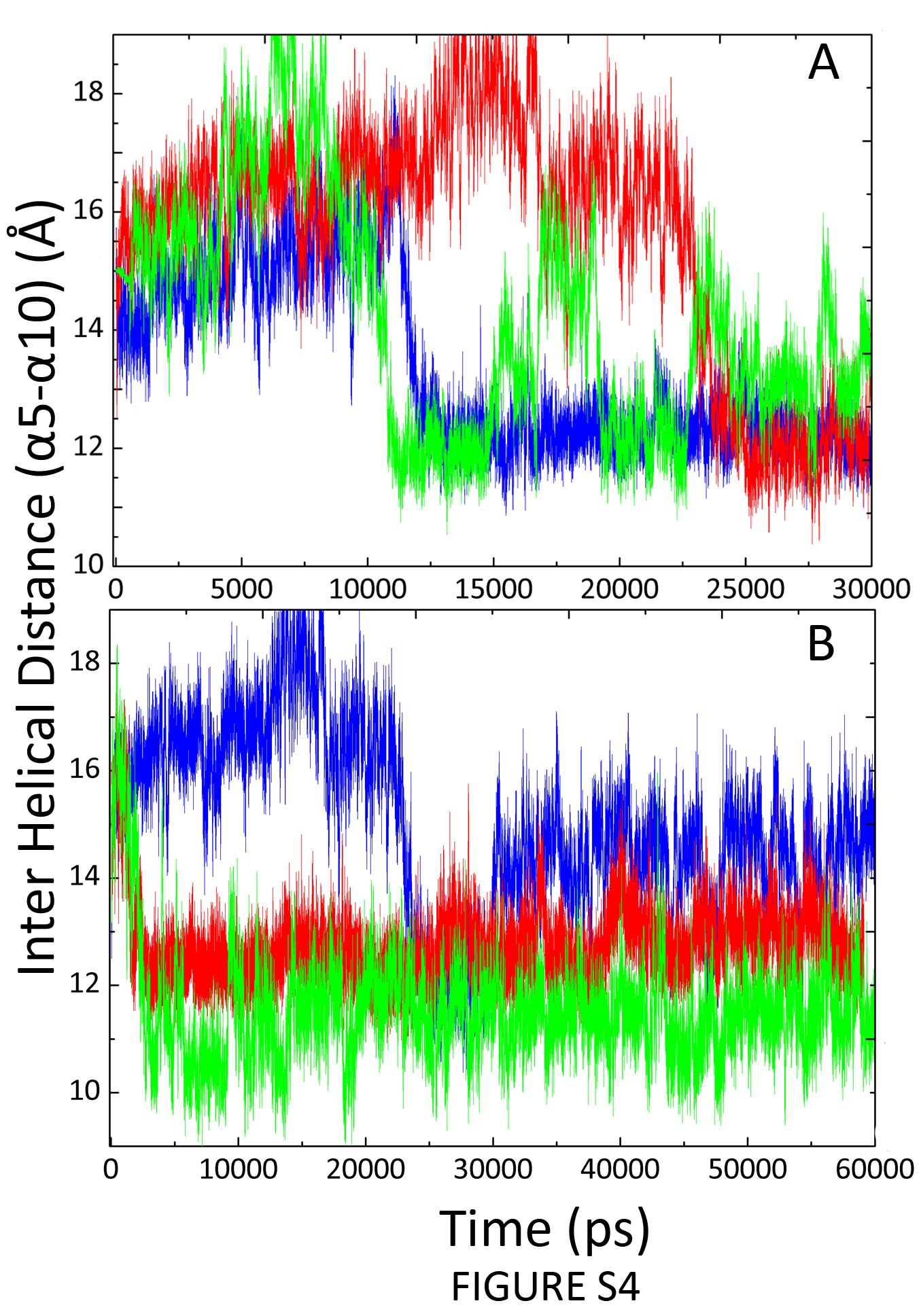

Supplement: Figure S4 — Distances between lid α5 and α10 at different sets of simulations. Inter helical distance (α5–α10) of three different simulations of CALB at 5°C for 30 ns. First experiment (blue), second experiment (red) and third experiment (green) (A). Inter helical distance of long–term simulation of CALB at 5°C (blue), 35°C (red) and 50°C (green) for 60 ns. Active site conformation closed and re-opened at 23 and 31 ns of simulation sequentially at 5°C (B). (Non-smoothed version of Figure 7) (TIF) [file pone.0040327.s004.tif]

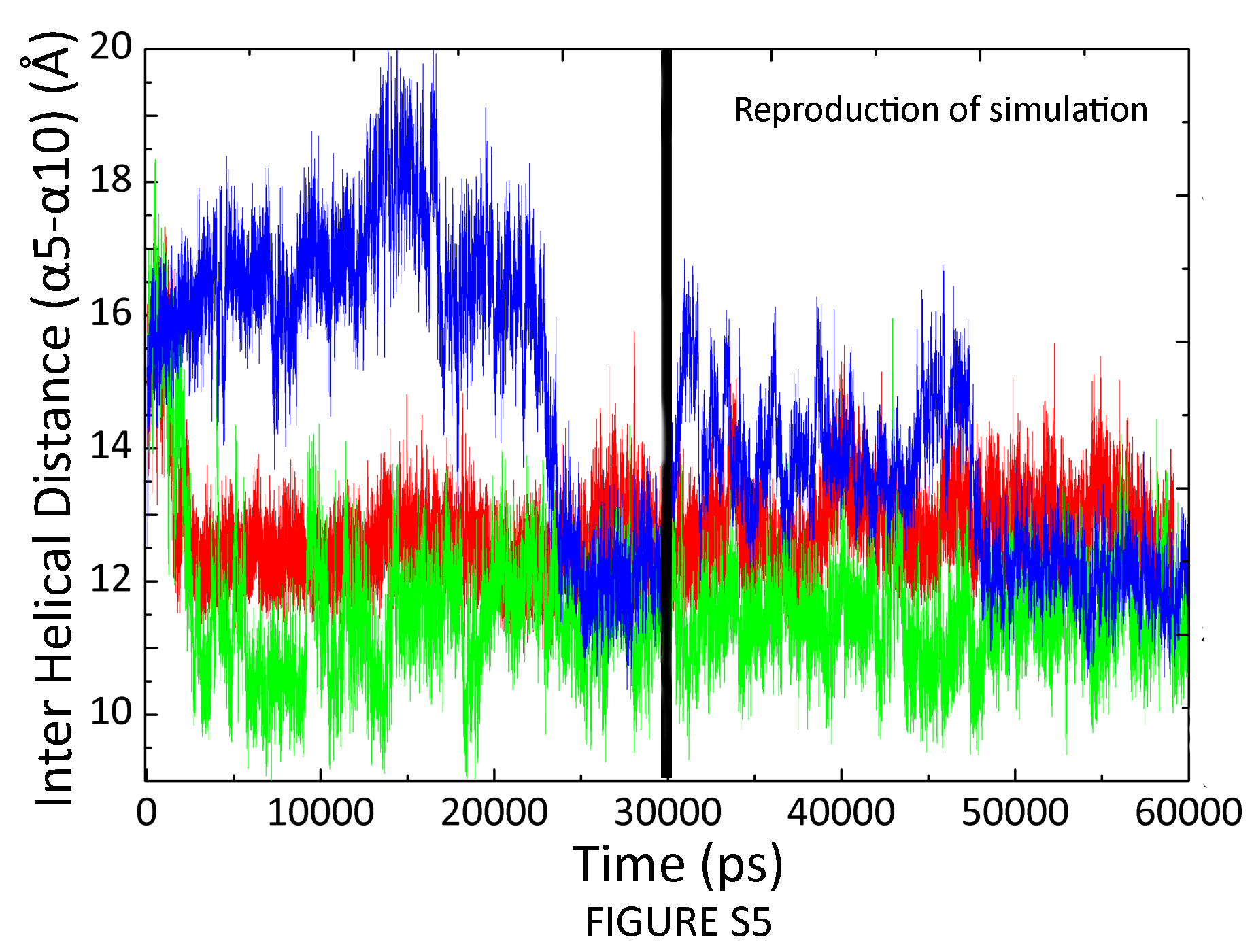

Supplement: Figure S5 — Distances between lid α5 and α10 at different sets of reproduced simulations. Inter helical distance (α5–α10) of reproduced long–term simulations of CALB at 5°C (blue), 35°C (red) and 50°C (green) for 60 ns. Simulations have been reproduces from 30 ns up to 60 ns. Active site conformation re-opened and closed at 32 ns and 47 ns of simulation at 5°C. (TIF) [file pone.0040327.s005.tif]

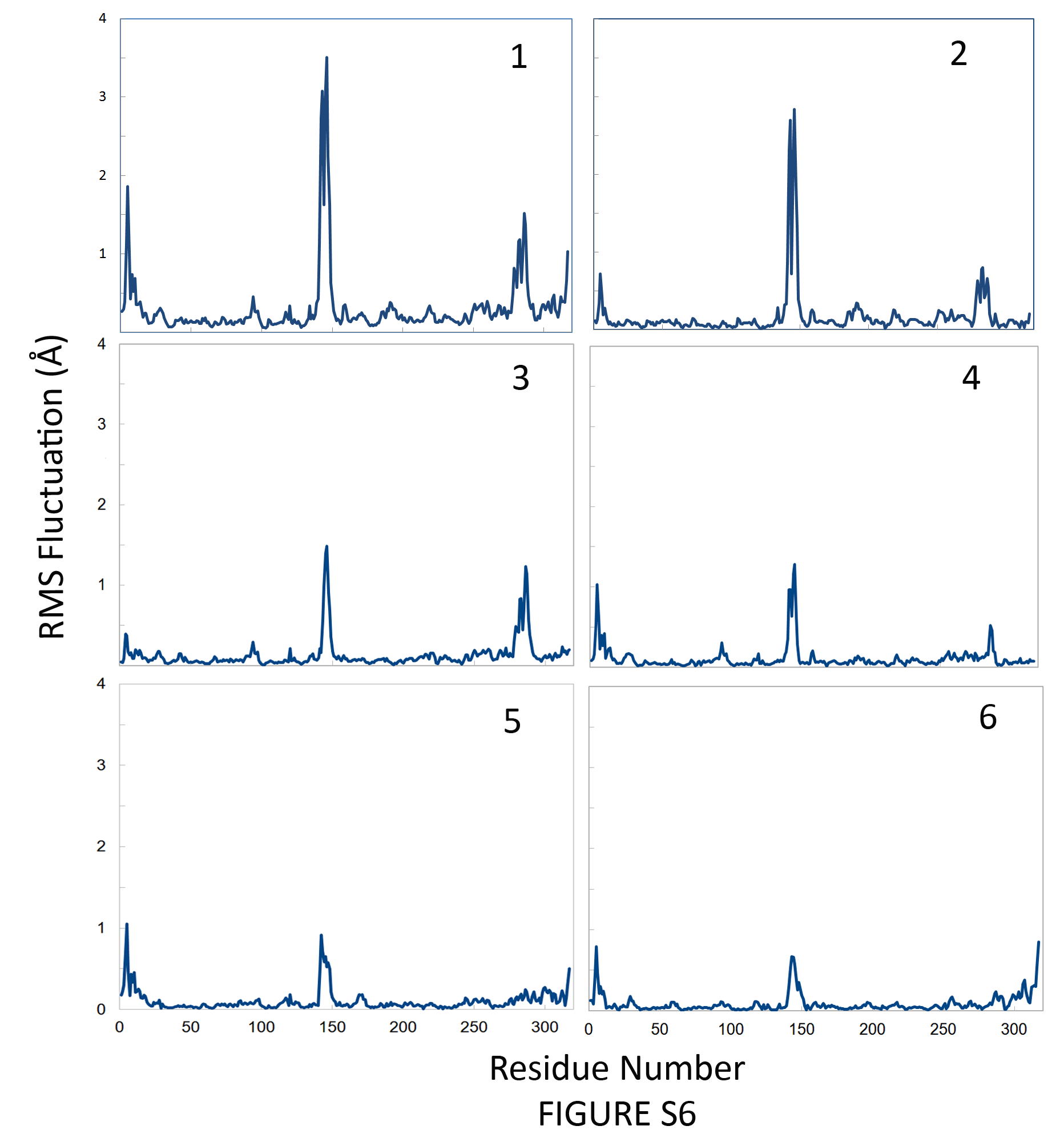

Supplement: Figure S6 — RMSF of CALB calculated from PCA at 5°C. RMS Fluctuations of CALB calculated from PCA for modes 1–5 at 5°C. Total RMSF (1), mode 1 (2), mode 2 (3), mode 3 (4), mode 4 (5), mode 5 (6). (TIF) [file pone.0040327.s006.tif]

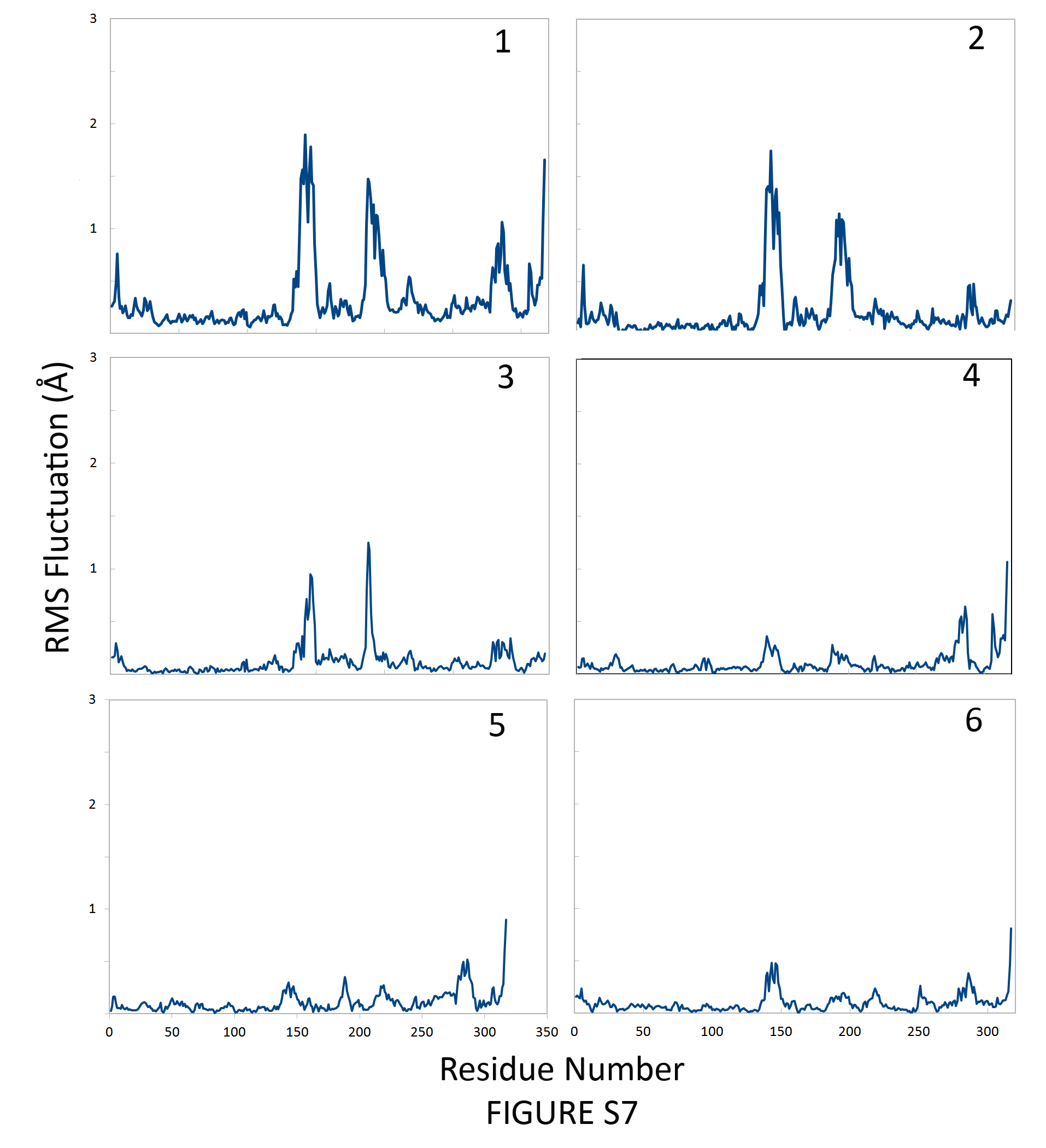

Supplement: Figure S7 — RMSF of CALB calculated from PCA at 35°C. RMS Fluctuations of CALB calculated from PCA for modes 1–5 at 35°C. Total RMSF (1), mode 1 (2), mode 2 (3), mode 3 (4), mode 4 (5), mode 5 (6). (TIF) [file pone.0040327.s007.tif]

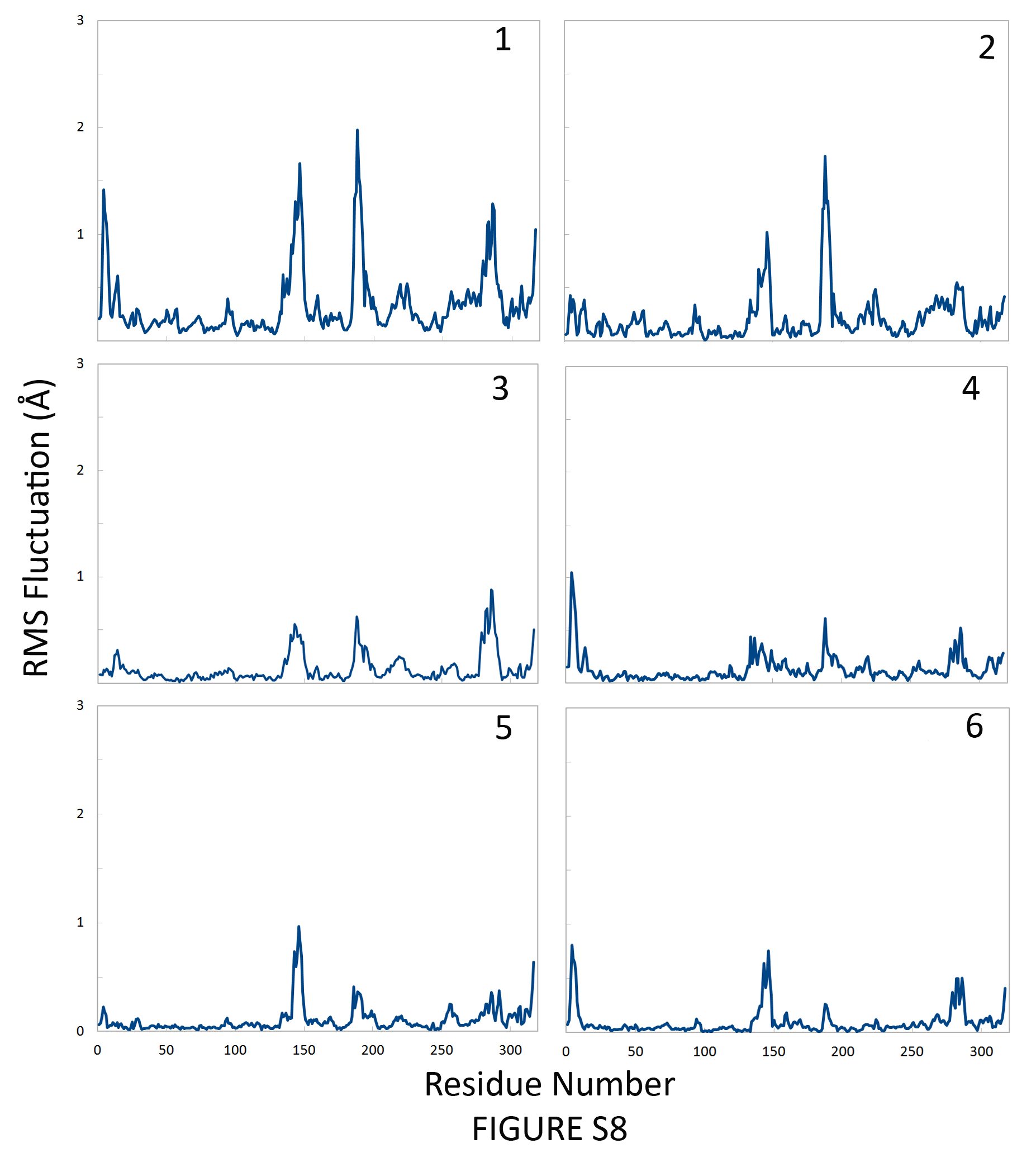

Supplement: Figure S8 — RMSF of CALB calculated from PCA at 50°C. RMS Fluctuations of CALB calculated from PCA for modes 1–5 at 50°C. Total RMSF (1), mode 1 (2), mode 2 (3), mode 3 (4), mode 4 (5), mode 5 (6). (TIF) [file pone.0040327.s008.tif]

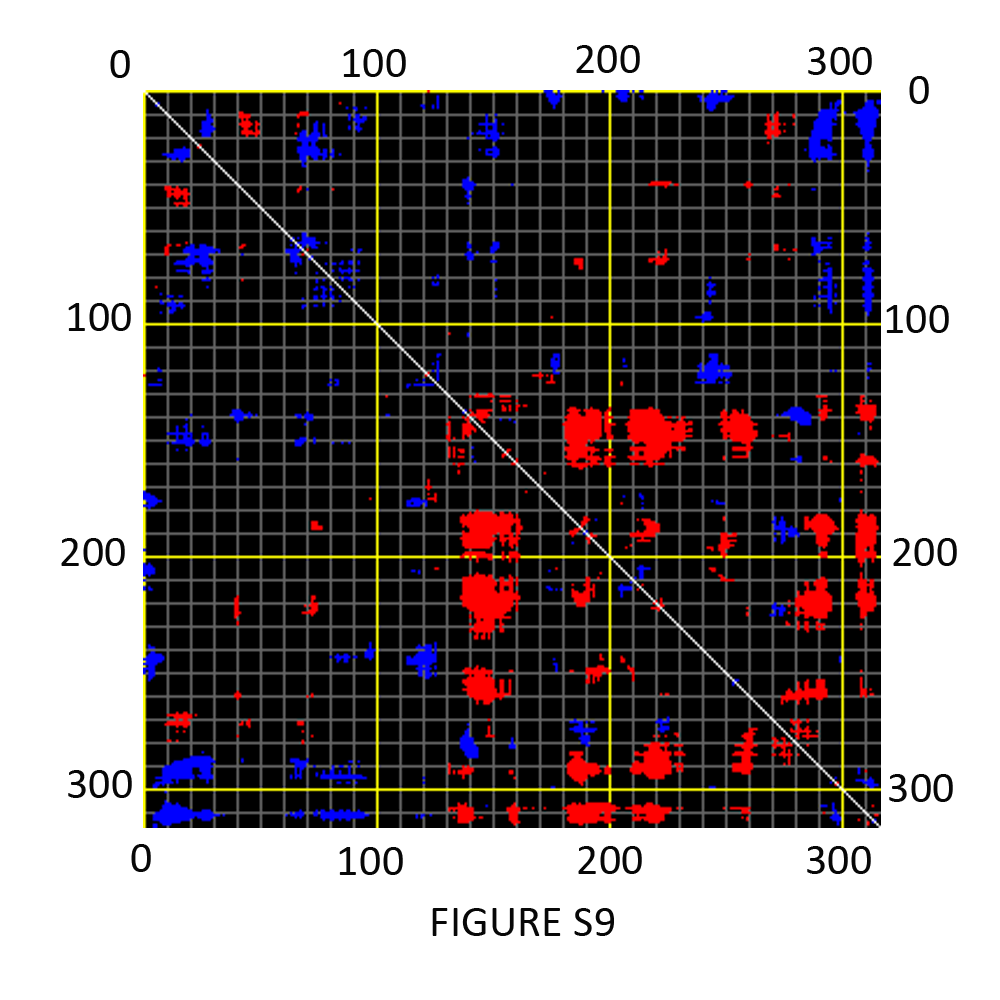

Supplement: Figure S9 — Distance Fluctuation map calculated from NMA. Distance Fluctuation map of mode 9 calculated from NMA. (TIF) [file pone.0040327.s009.tif]

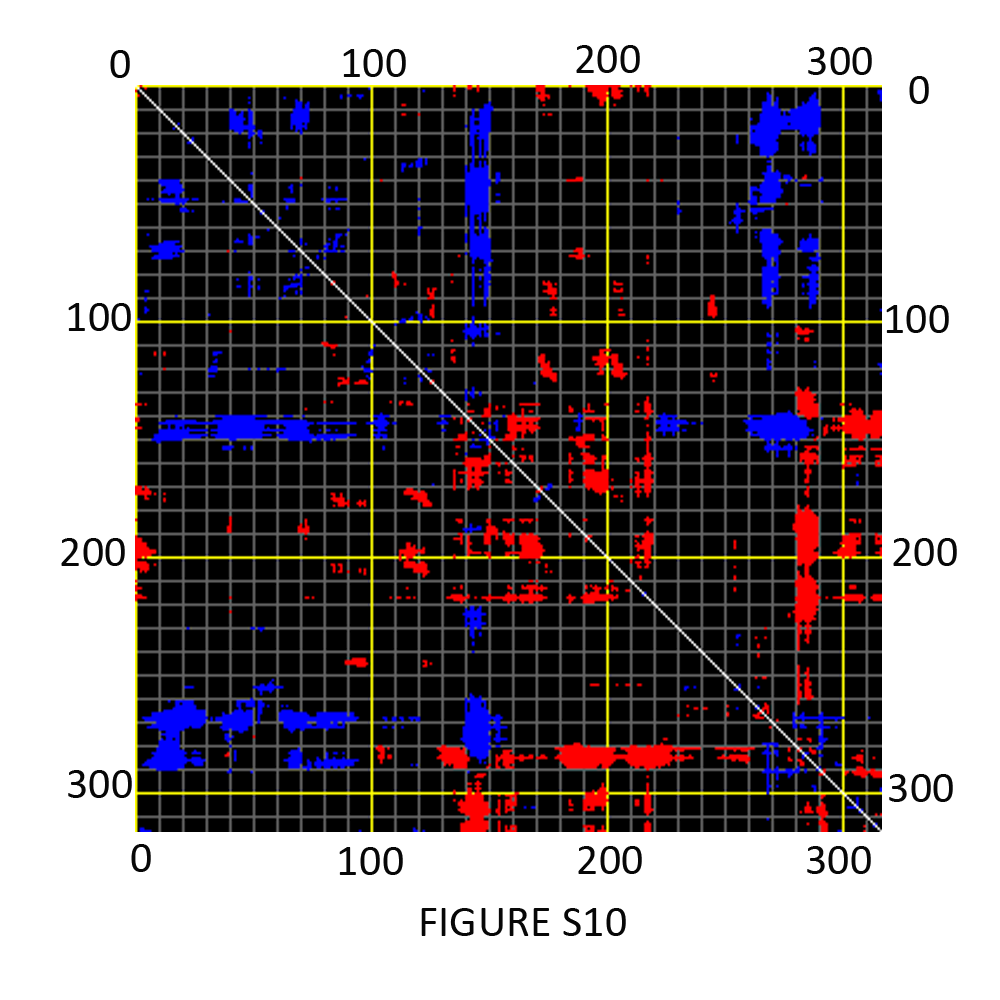

Supplement: Figure S10 — Distance Fluctuation map calculated from NMA. Distance Fluctuation map of mode 11 calculated from NMA. (TIF) [file pone.0040327.s010.tif]

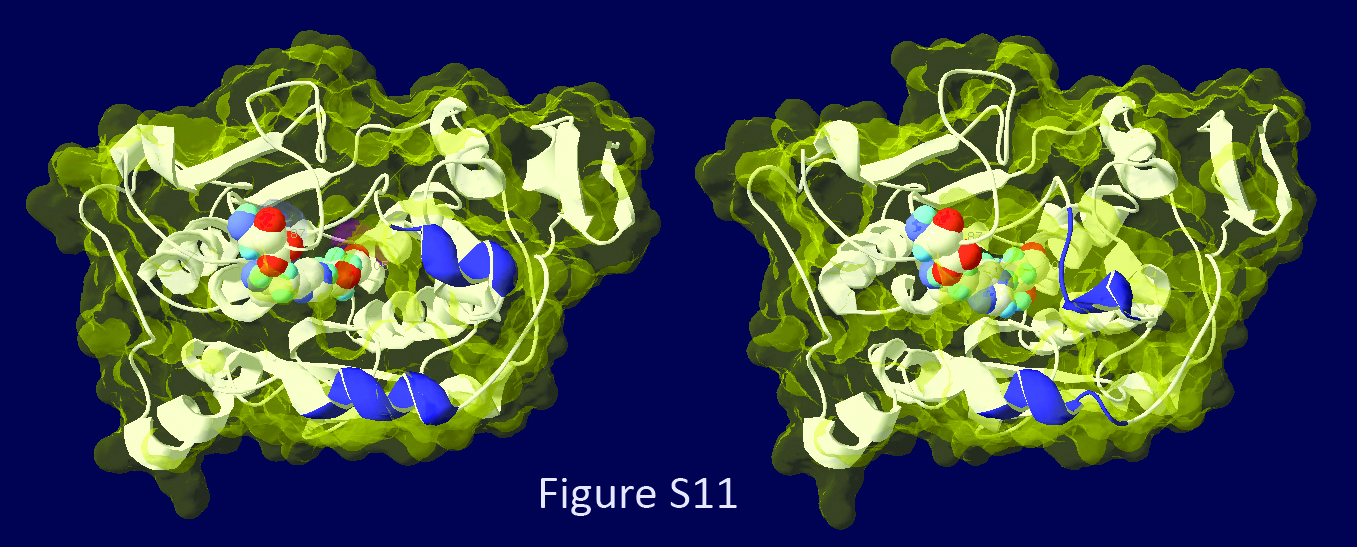

Supplement: Figure S11 — Ribbon representations of CALB. Ribbon representations of CALB at open (left) and closed (right) conformation. α5 (up) and α10 (down) are shown in blue. Semi-disordered α5 is clearly depicted in the closed conformation. (TIF) [file pone.0040327.s011.tif]

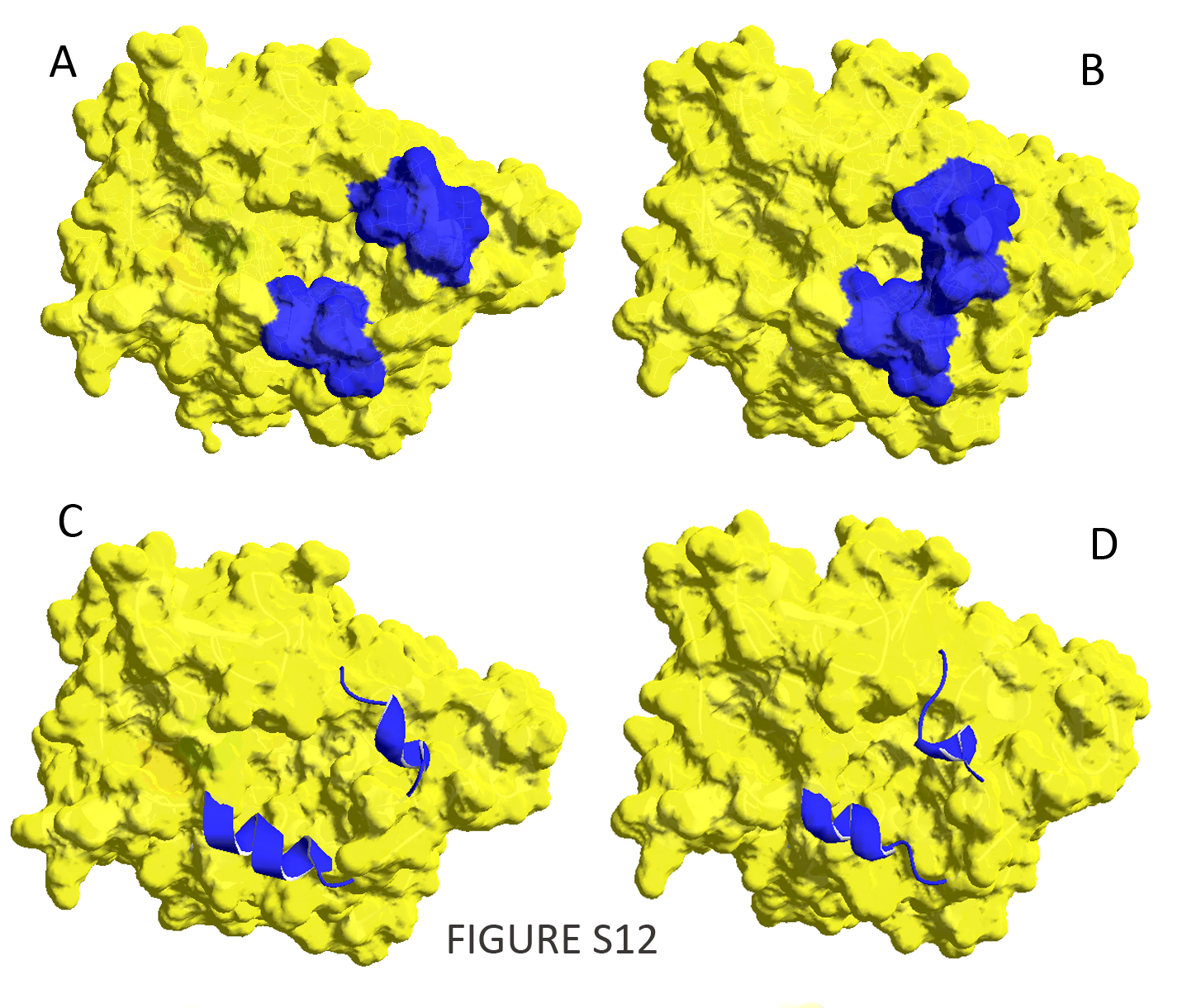

Supplement: Figure S12 — Surface and excluded ribbon representations of CALB at open and closed conformations. Surface representations of CALB at open and closed conformations (A and B). Excluded ribbon represents α5 and α10 in open and closed conformations (C and D). (TIF) [file pone.0040327.s012.tif]
